# A nationwide, population‐based study on specialized care for acute heart failure throughout the COVID‐19 pandemic

**DOI:** 10.1002/ejhf.3306

**Published:** 2024-06-04

**Authors:** Antonio Cannata, Mehrdad A. Mizani, Daniel I. Bromage, Susan E. Piper, Suzanna M.C. Hardman, Cathie Sudlow, Mark de Belder, John Deanfield, Roy S. Gardner, Andrew L. Clark, John G.F. Cleland, Theresa A. McDonagh, on behalf of the CVD‐COVID‐UK/COVID‐IMPACT Consortium

**Affiliations:** ^1^ British Heart Foundation Centre of Research Excellence, School of Cardiovascular Medicine, Faculty of Life Science King's College London London UK; ^2^ Cardiology Department King's College Hospital NHS Foundation Trust London UK; ^3^ British Heart Foundation Data Science Centre Health Data Research UK London UK; ^4^ Whittington Health London UK; ^5^ National Institute for Cardiovascular Outcomes Research (NICOR) NHS Arden and Greater East Midlands Commissioning Support Unit Leicester UK; ^6^ BHF Cardiovascular Research Centre, Institute of Cardiovascular and Medical Sciences University of Glasgow Glasgow UK; ^7^ Scottish National Advanced Heart Failure Service Golden Jubilee National Hospital Clydebank UK; ^8^ Hull University Teaching Hospitals Trust Hull UK; ^9^ British Heart Foundation Centre of Research Excellence, School of Cardiovascular and Metabolic Health University of Glasgow Glasgow UK

**Keywords:** Heart failure, Specialist care, COVID‐19, National Heart Failure Audit

## Abstract

**Aims:**

The COVID‐19 pandemic disrupted the delivery of care for patients with heart failure (HF), leading to fewer HF hospitalizations and increased mortality. However, nationwide data on quality of care and long‐term outcomes across the pandemic are scarce.

**Methods and results:**

We used data from the National Heart Failure Audit (NHFA) linked to national records for hospitalization and deaths. We compared pre‐COVID (2018–2019), COVID (2020), and late/post‐COVID (2021–2022) periods. Data for 227 250 patients admitted to hospital with HF were analysed and grouped according to the admission year and the presence of HF with (HFrEF) or without reduced ejection fraction (non‐HFrEF). The median age at admission was 81 years (interquartile range 72–88), 55% were men (*n* = 125 975), 87% were of white ethnicity (*n* = 102 805), and 51% had HFrEF (*n* = 116 990). In‐hospital management and specialized cardiology care were maintained throughout the pandemic with an increasing percentage of patients discharged on disease‐modifying medications over time (*p* < 0.001). Long‐term outcomes improved over time (hazard ratio [HR] 0.92, 95% confidence interval [CI] 0.90–0.95, *p* < 0.001), mainly driven by a reduction in cardiovascular death. Receiving specialized cardiology care was associated with better long‐term outcomes both for those who had HFrEF (HR 0.79, 95% CI 0.77–0.82, *p* < 0.001) and for those who had non‐HFrEF (HR 0.87, 95% CI 0.85–0.90, *p* < 0.001).

**Conclusions:**

Despite the disruption of healthcare systems, the clinical characteristics of patients admitted with HF were similar and the overall standard of care was maintained throughout the pandemic. Long‐term survival of patients hospitalized with HF continued to improve after COVID‐19, especially for HFrEF.

## Graphical abstract

The impact of the COVID‐19 pandemic on patients hospitalized with heart failure (HF) and the protective role of specialized care across the entire left ventricular ejection fraction spectrum. CI, confidence interval; HFrEF, heart failure with reduced ejection fraction; HR, hazard ratio.

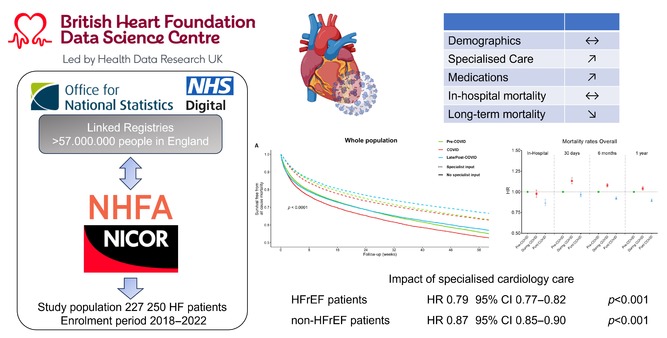

## Introduction

Hospitalization for heart failure (HF) is associated with a high inpatient mortality and approximately one third die in the following year.^1^, ^2^ However, comprehensive guideline‐recommended medical therapy (GRMT) for patients with HF with reduced ejection fraction (HFrEF) may extend life expectancy two‐ to three‐fold compared to historical conventional therapy.^3^

The coronavirus disease 2019 (COVID‐19) pandemic has been a dramatic global public health emergency. The impact and the pressure on healthcare systems caused by the COVID‐19 pandemic led to a significant reconfiguration, including staff redeployment and a reduction in routine appointments for stable chronic conditions.^4^, ^5^, ^6^ Early reports, predominantly from the first wave of the pandemic, estimated a three‐fold excess mortality in patients with established cardiovascular disease.^4^, ^5^, ^6^ For patients with HF in the UK, there was a 47% fall in hospital admissions for HF during the first wave of the pandemic.^7^ Patients who were admitted appeared to be generally sicker, and several aspects of in‐hospital management such as place of care and therapy differed from previous years.^8^, ^9^, ^10^ These changes were associated with a significant increase both for in‐hospital mortality and deaths in the community during the peak of the pandemic for patients with HF.^10^, ^11^, ^12^, ^13^ Conversely, in countries where the impact of the pandemic was lower, such as Denmark, an increase in cardiovascular mortality was avoided, perhaps because specialist care was preserved.^14^ However, many reports available are from single centres. Nationwide analyses on the impact of the COVID‐19 pandemic are limited.^15^, ^16^, ^17^

The aim of this analysis is to describe the impact of the COVID‐19 pandemic on specialist care for patients hospitalized with HF and related outcomes at a national level using data collected over 5 years before, during and after the height of the COVID‐19 pandemic.

## Methods

### Data sources

The British Heart Foundation Data Science Centre enabled access to key national datasets made available for the CVD‐COVID‐UK/COVID‐IMPACT Consortium (https://bhfdatasciencecentre.org/areas/cvd‐covid‐uk‐covid‐impact/) via NHS England's Secure Data Environment service for England, that is: the National Heart Failure Audit (NHFA), Hospital Episode Statistics (HES) Admitted Patient Care (APC), and the Office for National Statistics (ONS) Civil Registrations of Death Registry, from which we derived the cohort described here. The NHFA, one of the domains of the National Cardiac Audit Programme, is a prospective audit of hospital admissions designed to assess the quality of care for hospitalized patients with HF in England and Wales (https://www.nicor.org.uk/national‐cardiac‐audit‐programme/heart‐failure‐audit‐nhfa). It reports data on HF hospitalizations from NHS Trusts in England and Health Boards in Wales. Case ascertainment is over 80%.^1^, ^7^ Data entry is mandatory for all NHS trusts admitting patients with acute HF. Patients are entered into the audit if they have a primary diagnosis of HF at discharge or death for those who did not survive to discharge. Mandatory fields in the NHFA include demographics, presenting symptoms and signs, comorbidities, diagnostic tests, specialist cardiology input, length of stay, prescribed medications, and follow‐up arrangements. We used de‐identified individual patient data from the NHFA linked with national administrative registries, namely the HES and ONS databases. Linkage of key variables provided the most complete and reliable information possible for each patient, as previously described.^18^, ^19^, ^20^ Datasets were linked at the individual level by NHS England using a pseudonymized version of the NHS number. To identify HF diagnoses, we used a list of 107 diagnostic codes (International Classification of Diseases, tenth revision [ICD‐10]) from the hospital coding scheme (HES‐APC) and NHFA.^18^, ^19^, ^20^

Over the 5 years of data collection, the phenotypic classification of HF according to left ventricular ejection fraction (LVEF) changed, as did the definitions in the NHFA.^2^, ^21^ Thus, we pragmatically assigned patients into two phenotypic groups based on LVEF. We classified HFrEF as HF and significant left ventricular systolic dysfunction, defined as LVEF ≤40%. Conversely, patients admitted because of HF with evidence of a cardiac structural or functional abnormality in the absence of significant systolic dysfunction (i.e. LVEF >40%) were defined as non‐HFrEF. We chose not to label this as HF with preserved ejection fraction as we were unable to apply the European Society of Cardiology criteria for that definition and the group also includes patients with significant valve disease (see online supplementary *Methods* for more details).

### Events of interest

We defined the COVID period as the year in which the first COVID case occurred in the UK (1 January 2020–31 December 2020). We then compared events of interest in the 2 years before (pre‐COVID; 1 January 2018–31 December 2019) and the 2 years after (late/post‐ COVID; 1 January 2021–31 December 2022). We chose the late/post‐COVID time frame from January 2021 for consistency with the other time frames. Although there were continuing cases of COVID‐19 in January and February, all lockdown measures were eased from the beginning of 2021, and the vaccination rollout commenced in December 2020 and accelerated from January 2021. We used 2‐year comparison periods before and after the COVID‐19 pandemic to avoid the effects of potential seasonal variations. Furthermore, to increase the granularity of the analysis within the COVID period, we performed a month‐by‐month analysis subdividing according to the main lockdown measures. If the patients had more than one admission, only the first admission per patient was included in the analysis.

### Quality of care

We assessed quality of care as the proportion of hospitalized patients receiving specialist cardiology care during admission, as recommended by the National Institute for Care and Excellence.^22^, ^23^, ^24^ Specialist cardiology care was defined as the referral to, and subsequent consultation with, either a cardiologist or a HF specialist during the index admission, as reported in the NHFA. Furthermore, we evaluated the prescription rates for medical therapy for HF and follow‐up arrangements at discharge.

### Outcome measures

The primary outcome measure was all‐cause mortality. Secondary outcomes were cause‐specific death rates and in‐hospital mortality. We obtained mortality rates and causes of death through linkage to the ONS database reporting the causes of death recorded on death certificates. In‐hospital mortality was defined as death occurring during the index admission spell.

### Sensitivity analyses

We performed the following sensitivity analysis: reclassifying the definition of HF, including those with LVEF between 41% and 50% (i.e. HF with mildly reduced ejection fraction) in the HFrEF group; and excluding patients enrolled in 2022 who may not have accrued sufficient follow‐up.

### Statistical analysis

Comparisons between the time‐period groups were made by the Mann–Whitney U test and the chi‐square test, where appropriate. Continuous variables are reported as medians (25th–75th centiles). Categorical variables are reported as numbers (percentages). Incidence rate ratios (IRR) comparing the different periods were calculated using the Poisson regression to model the number of weekly hospitalizations for HF. Survival curves for all‐cause mortality were estimated and compared between groups by means of the log‐rank test. Cause‐specific death rates were estimated and compared using the Fine–Gray test for competing risk.^25^ To adjust for confounders, we used a multivariable Cox proportional hazards model with a list of candidate prognostic variables including demographic characteristics, symptoms and signs of HF and severity of illness at presentation. We assessed the proportional hazards assumption using the Schoenfeld residual test. We also used a piecewise hazards model at 30 days, 6 months and 1 year applying a Cox proportional hazards model within each period to hold the proportional hazard assumption.^26^ A *p*‐value of ≤ 0.10 was the entry threshold for the multivariable analysis. In all multivariable methods, we conducted a complete case analysis removing variables with significant amounts of missing data (i.e. >10%). We also handled missing data for variables of interest by multiple imputation with chained equations and performed the analysis on the imputed dataset. A *p*‐value of < 0.01 was considered statistically significant. All analyses were conducted using ‘R’ (Version 4.2.2, R Foundation for Statistical Computing, Vienna, Austria; https://www.r‐project.org/) according to a pre‐specified analysis plan published on GitHub (https://github.com/BHFDSC/CCU045_01.git). De‐identified data were accessed through secure remote access to NHS England's SDE.

## Results

### Patient characteristics

The population included 227 250 patients and 299 040 HF admissions. Of these, 104 560 patients were admitted pre‐COVID, corresponding to 1529 admissions per week, 44 020 during COVID (1347 admissions per week), and 78 670 late/post‐COVID (1520 admissions per week). The relative risk of admission was approximately 1% lower during COVID (IRR 0.99, CI 0.99–1.00, *p* = 0.05) with a similar rebound late/post‐COVID (IRR 1.01, 95% CI 1.00–1.01, *p* < 0.001) (online supplementary *Figure* *S1*).

Patient characteristics are reported in *Tables* *1* and *2*. Overall, 51% of patients had HFrEF (*n* = 116 990), while the rest had non‐HFrEF. Among patients with HFrEF, 57 280 were admitted pre‐COVID, 23 280 during COVID and 36 430 late/post‐COVID. Among patients admitted with non‐HFrEF, 47 280 were admitted pre‐COVID, 20 740 during COVID and 42 240 late/post‐COVID.

**Table 1:** Characteristics of the overall study cohort

	Pre‐COVID (2018–2019)	COVID (2020)	Late/post‐COVID (2021–2022)
Patients, *n*	104 560	44 020	78 670
Male sex	57 700 (55)	24 320 (55)	43 955 (56)
Age at admission, years	81 [73–88]	81 [72–88]	81 [72–87]
Ethnicity			
White	45 475 (86)	19 935 (89)	35 395 (86)
Black	1470 (3)	580 (3)	1205 (3)
Asian	3275 (6)	1100 (5)	1430 (4)
Other	2550 (5)	790 (4)	2310 (6)
NYHA class III/IV	77 780 (78)	32 440 (78)	57 555 (77)
Moderate or severe oedema	52 420 (54)	21 800 (53)	38 660 (53)
Comorbidity			
IHD	39 440 (39)	15 235 (36)	25 225 (33)
Pre‐existing valve disease	30 265 (30)	12 210 (29)	21 010 (27)
Hypertension	57 710 (56)	25 045 (58)	45 650 (59)
Diabetes	34 540 (34)	14 080 (33)	25 405 (33)
Respiratory disease	25 170 (25)	10 540 (25)	18 695 (24)
ECG			
Sinus	38 710 (39)	15 885 (38)	30 325 (41)
Atrial fibrillation	50 370 (51)	21 705 (52)	37 990 (51)
Other	9895 (10)	4005 (9)	6035 (8)
Physical examination			
Heart rate, bpm	84 [70–100]	85 [71–102]	85 [71–101]
Systolic BP, mmHg	131 [115–150]	133 [116–153]	133 [116–152]
Blood tests			
Creatinine at admission, mg/dl	105 [82–144]	104 [81–141]	103 [80–139]
eGFR (CKD‐EPI), ml/min/1.73 m^2^	50 [34–69]	51 [34–70]	52 [36–72]
Potassium, mEq/L	4.2 [3.8–4.6]	4.2 [3.8–4.5]	4.2 [3.8–4.5]
HF types			
HFrEF	57 280 (55)	23 280 (53)	36 430 (46)
Non‐HFrEF	47 280 (45)	20 740 (47)	42 240 (54)
Discharge medications			
RASi/ARNI	45 790 (63)	19 690 (67)	37 490 (64)
Beta‐blocker	60 560 (79)	26 075 (80)	49 030 (81)
Diuretics	74 290 (92)	31 160 (91)	57 100 (90)
MRAs	27 745 (43)	12 025 (45)	24 170 (47)
Nurse follow‐up	46 430 (56)	18 315 (52)	21 195 (32)
Cardiology follow‐up	39 665 (48)	15 550 (45)	22 000 (34)
Specialist input	82 800 (80)	35 095 (80)	64 865 (82)
Follow‐up (weeks)	105 [21–190]	105 [19–129]	43 [19–71]
Length of stay (days)	8 [4–14]	7 [4–13]	7 [4–14]

Values are reported as *n* (%), or median [25th–75th centiles].

ARNI, angiotensin receptor–neprilysin inhibitor; BP, blood pressure; CKD‐EPI, Chronic Kidney Disease Epidemiology Collaboration; ECG, electrocardiogram; eGFR, estimated glomerular filtration rate; HFrEF, heart failure with reduced ejection fraction; IHD, ischaemic heart disease; MRA, mineralocorticoid receptor antagonist; NYHA, New York Heart Association; RASi, renin–angiotensin system inhibitor.

**Table 2:** Characteristics of the heart failure with reduced ejection fraction (HFrEF) and non‐HFrEF cohorts

	HFrEF			Non‐HFrEF		
	Pre‐COVID (2018–2019)	COVID (2020)	Late/post‐COVID (2021–2022)	Pre‐COVID (2018–2019)	COVID (2020)	Late/post‐COVID (2021–2022)
Patients, *n*	57 280	23 280	36 430	47 280	20 740	42 240
Male sex	36 700 (64)	14 910 (64)	25 770 (65)	21 000 (44)	9410 (45)	18 185 (47)
Age at admission	79 [70–86]	78 [68–86]	78 [67–86]	86 [76–89]	83 [76–86]	83 [76–89]
Ethnicity						
White	25 650 (86)	10 330 (89)	17 920 (90)	19 825 (86)	9605 (89)	19 475 (87)
Black	840 (3)	325 (3)	660 (3)	630 (3)	255 (2)	545 (2)
Asian	1840 (6)	560 (5)	120 (1)	1435 (6)	540 (5)	1310 (6)
Other	1430 (5)	440 (4)	1260 (6)	1120 (5)	350 (3)	1050 (5)
NYHA class III/IV	42 455 (77)	17 030 (77)	26 600 (77)	35 325 (77)	15 410 (78)	30 955 (77)
Moderate or severe oedema	26 420 (49)	10 430 (48)	15 080 (46)	26 000 (49)	11 370 (59)	23 580 (58)
IHD	24 250 (44)	8840 (40)	12 820 (36)	15 190 (33)	6395 (32)	12 405 (30)
Pre‐existing valve disease	15 080 (27)	5690 (25)	8830 (23)	15 185 (33)	6520 (33)	12 180 (30)
Hypertension	29 220 (52)	12 155 (53)	19 135 (54)	28 490 (61)	12 890 (63)	26 515 (64)
Diabetes	18 940 (34)	7350 (32)	11 460 (32)	15 600 (34)	6730 (33)	13 945 (33)
Respiratory disease	13 130 (24)	5240 (23)	7815 (22)	12 040 (31)	5300 (26)	10 880 (26)
ECG						
Sinus	24 490 (46)	9900 (46)	16 925 (50)	14 220 (31)	5985 (30)	13 400 (33)
Atrial fibrillation	22 240 (42)	9180 (42)	13 590 (40)	28 130 (62)	12 525 (63)	24 400 (60)
Other	6640 (12)	2565 (12)	3375 (10)	3255 (7)	1440 (7)	2660 (7)
Physical examination						
Heart rate, bpm	86 [72–104]	89 [74–107]	89 [74–107]	81 [69–97]	82 [70–98]	82 [69–96]
Systolic BP, mmHg	128 [112–147]	130 [113–149]	130 [113–148]	135 [118–154]	137 [119–156]	136 [119–156]
Creatinine at admission, mg/dl	107 [83–146]	105 [82–141]	103 [81–139]	104 [80–141]	103 [79–141]	102 [79–139]
eGFR (CKD‐EPI), ml/min/1.73 m^2^	51 [34–70]	53 [36–72]	55 [37–75]	49 [34–67]	50 [33–67]	50 [34–68]
Potassium, mEq/L	4.2 [3.9–4.6]	4.2 [3.8–4.6]	4.2 [3.9–4.6]	4.2 [3.8–4.6]	4.1 [3.8–4.5]	4.1 [3.8–4.5]
Discharge medications						
RASi/ARNI	30 350 (70)	12 920 (72)	21 930 (74)	15 440 (53)	6770 (53)	15 560 (54)
Beta‐blocker	36 980 (84)	15 515 (85)	25 620 (86)	23 580 (73)	10 560 (74)	23 410 (76)
Diuretics	39 920 (90)	16 000 (89)	25 120 (87)	34 370 (93)	15 160 (93)	31 980 (93)
MRAs	19 685 (51)	8470 (54)	15 900 (59)	8060 (32)	3555 (33)	8270 (35)
Nurse follow‐up	33 130 (73)	12 975 (69)	13 635 (45)	13 300 (36)	5340 (33)	7560 (22)
Cardiology follow‐up	25 775 (58)	9910 (54)	12 340 (41)	13 890 (37)	5640 (35)	9660 (28)
Specialist input	49 740 (86)	20 555 (88)	33 265 (91)	33 060 (70)	14 540 (70)	31 600 (75)
Follow‐up (weeks)	122 [23–196]	110 [21–131]	45 [20–73]	90 [20–181]	85 [15–125]	42 [18–69]
Length of stay (days)	8 [4–15]	7 [4–13]	8 [4–14]	7 [3–14]	7 [3–13]	7 [3–13]

Values are reported as *n* (%), or median [25th–75th centiles].

ARNI, angiotensin receptor–neprilysin inhibitor; BP, blood pressure; CKD‐EPI, Chronic Kidney Disease Epidemiology Collaboration; ECG, electrocardiogram; eGFR, estimated glomerular filtration rate; HFrEF, heart failure with reduced ejection fraction; IHD, ischaemic heart disease; MRA, mineralocorticoid receptor antagonist; NYHA, New York Heart Association; RASi, renin–angiotensin system inhibitor.

The demographic characteristics of the patients and comorbidity profiles were similar in all three periods of interest, as well as between non‐HFrEF and HFrEF cohorts. Most patients were men (55%, *n* = 125 975) and of white ethnicity (87%, *n* = 102 805). Patients admitted with non‐HFrEF were more commonly women (56%, *n* = 61 665 for non‐HFrEF vs. 44%, *n* = 39 610 for HFrEF) and older compared to HFrEF (83 years of age for non‐HFrEF vs. 79 years of age for HFrEF). Signs and symptoms of HF were similar across the three periods of interest and between the HFrEF and non‐HFrEF patients.

### Aetiology of heart failure

The aetiology of HF was non‐ischaemic in approximately 64% of cases (*n* = 147 350), and 29% of patients had a history of moderate‐to‐severe valve disease (*n* = 63 485). Hypertension was present in 57% of patients (*n* = 128 405), while diabetes and respiratory disease were present in approximately 33% (*n* = 74 025) and 24% (*n* = 54 405), respectively.

### In‐hospital management and specialized cardiology care

Approximately 80% of patients with HF received specialized cardiology input during hospitalization. A higher proportion of patients received specialized cardiology input late/post‐ COVID compared to COVID and pre‐COVID (82% vs. 80% vs. 79%, respectively, *p* < 0.001). A total of 88% of patients with HFrEF received specialized cardiology input during hospitalization, while 72% of patients with non‐HFrEF were seen by a cardiologist. Both groups received more cardiology input over time (87% in pre‐COVID vs. 88% during COVID vs. 91% late/post‐COVID for HFrEF, *p* < 0.001; and 70% in pre‐COVID vs. 70% during COVID vs. 75% late/post‐COVID for non‐HFrEF, *p* < 0.001).

The proportion of patients with HFrEF on renin–angiotensin system inhibitors increased over time (70% pre‐COVID vs. 72% during COVID vs. 74% late/post‐COVID, *p* < 0.001), as did the proportion on beta‐blockers (84% pre‐COVID vs. 85% during COVID vs. 86% late/post‐COVID, *p* < 0.001) and mineralocorticoid receptor antagonists (51% pre‐COVID vs. 54% during COVID vs. 59% late/post‐COVID, *p* < 0.001), with an inverse trend in diuretic prescription at discharge (90% pre‐COVID vs. 89% during COVID vs. 87% late/post‐COVID, *p* < 0.001) (*Table* *2*).

The median length of stay in hospital was 8 (3–14) days pre‐COVID, falling to 7 (4–13) days during and late/post‐COVID (*p* < 0.001). The fall was mainly driven by a reduction in the length of stay in patients with HFrEF.

### Outcomes

**Figure 1:**
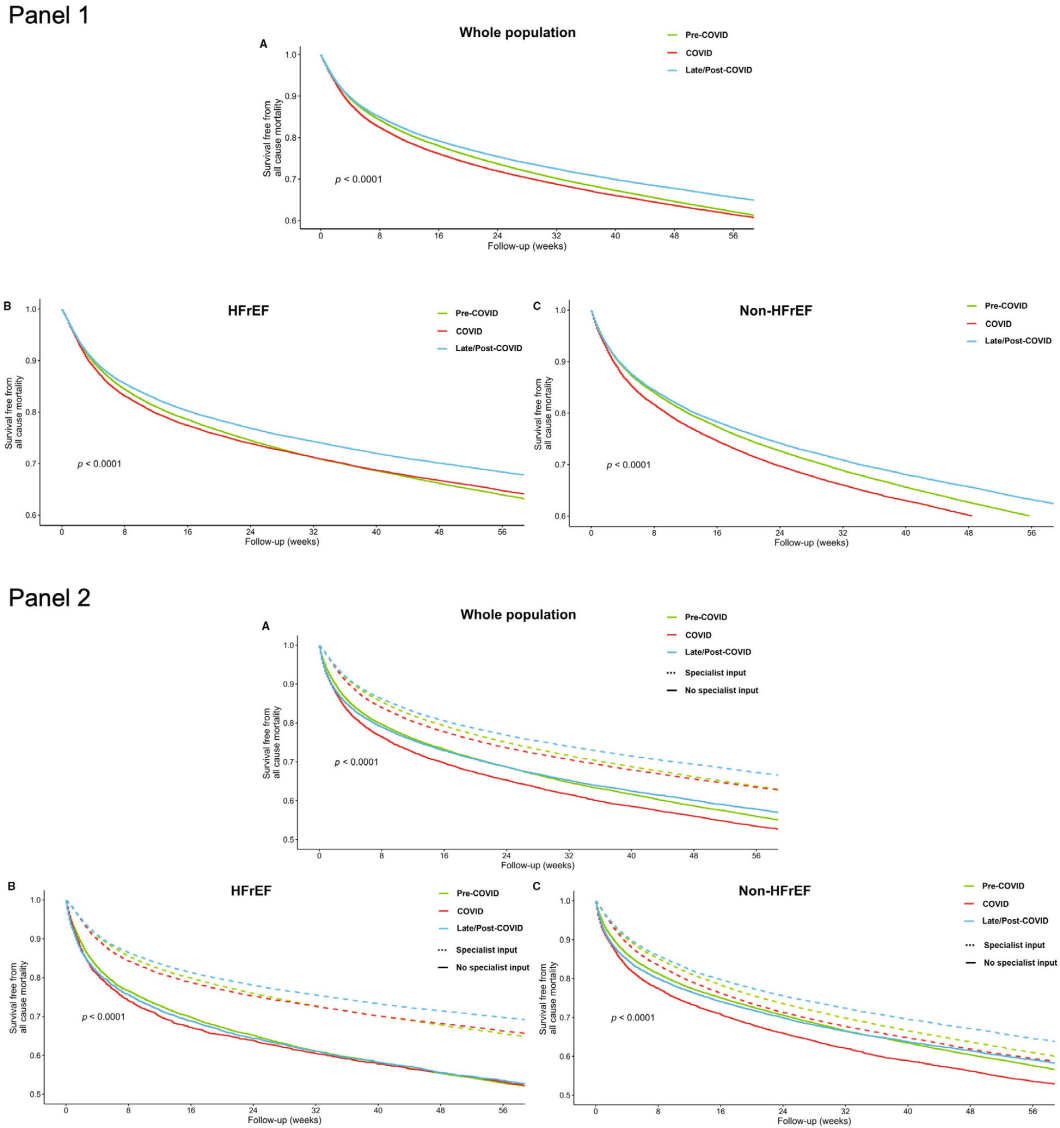
(*Panel 1*) Kaplan–Meier curves for overall survival from admission by period in the overall cohort of patients with heart failure (*A*), in patients with heart failure with reduced ejection fraction (HFrEF) (*B*) and in patients with non‐HFrEF (*C*). (*Panel 2*) Kaplan–Meier curves for overall survival from admission by period and the specialized cardiology care received in the overall cohort of patients with heart failure (*A*), in patients with HFrEF (*B*) and in patients with non‐HFrEF (*C*).

Compared to the pre‐COVID period, patients admitted because of HF during COVID or late/post‐COVID had a better longer‐term outcome (*p* < 0.001; *Figure* *1A*). Although patients admitted with HFrEF during COVID had an initially worse mortality in the early months after hospitalization compared to pre‐COVID, overall survival improved over time (*p* < 0.001; *Figure* *1B* and *Table* *3*). Conversely, patients with non‐HFrEF admitted during COVID had worse outcomes compared to pre‐ and late/post‐COVID (*p* < 0.001; *Figure* *1C* and *Table* *3*).

**Table 3:** Mortality rates and risk of adverse events at different time points

	Pre‐COVID		During‐COVID					Late/post‐COVID			
	*n* (%)		*n* (%)		HR^a^	95% CI	*p*‐value*	*n* (%)	HR^a^	95% CI	*p*‐value*
Overall											
In‐hospital	7925 (8.6)	ref	3300 (8.4)		0.98	0.93–1.02	0.20	5330 (7.4)	0.87	0.83–0.91	<0.001
30 days	11 185 (11)	ref	5300 (12)		1.13	1.10–1.17	<0.001	8180 (10)	0.97	0.94–1.00	0.046
6 months	28 550 (27)	ref	12 755 (29)		1.08	1.06–1.10	<0.001	19 820 (25)	0.93	0.91–0.94	<0.001
1 year	38 330 (37)	ref	16 495 (37)		1.04	1.02–1.06	<0.001	24 720 (31)	0.90	0.88–0.91	<0.001
HFrEF											
In‐hospital	4115 (8.2)	ref	1560 (7.5)		0.92	0.86–0.97	0.004	2240 (6.8)	0.81	0.77–0.86	<0.001
30 days	5995 (10)	ref	2635 (11)		1.07	0.98–1.17	0.11	3610 (10)	1.00	0.20–1.07	0.99
6 months	15 130 (26)	ref	6255 (27)		1.03	1.00–1.06	0.08	8615 (24)	0.89	0.87–0.92	<0.001
1 year	20 040 (35)	ref	7965 (34)		0.98	0.96–1.01	0.20	10 595 (29)	0.87	0.85–0.89	<0.001
Non‐HFrEF											
In‐hospital	3810 (9.1)	ref	1740 (9.0)		1.03	0.97–1.09	0.30	3090 (7.9)	0.86	0.82–0.90	<0.001
30 days	5230 (11)	ref	2670 (13)		1.15	1.07–1.24	<0.001	4570 (11)	1.05	0.99–1.12	0.13
6 months	13 420 (28)	ref	6500 (31)		1.13	1.10–1.16	<0.001	11 210 (27)	0.94	0.92–0.96	<0.001
1 year	18 290 (39)	ref	8530 (41)		1.09	1.07–1.12	<0.001	14 120 (33)	0.91	0.89–0.93	<0.001

CI, confidence interval; HFrEF, heart failure with reduced ejection fraction; HR, hazard ratio.

^a^ Odds ratio for in‐hospital mortality and HR for 30‐day, 6‐month and 1‐year mortality.

* Logistic regression for in‐hospital mortality and Cox‐proportional hazard model for 30‐day, 6‐month and 1‐year mortality.

**Figure 2:**
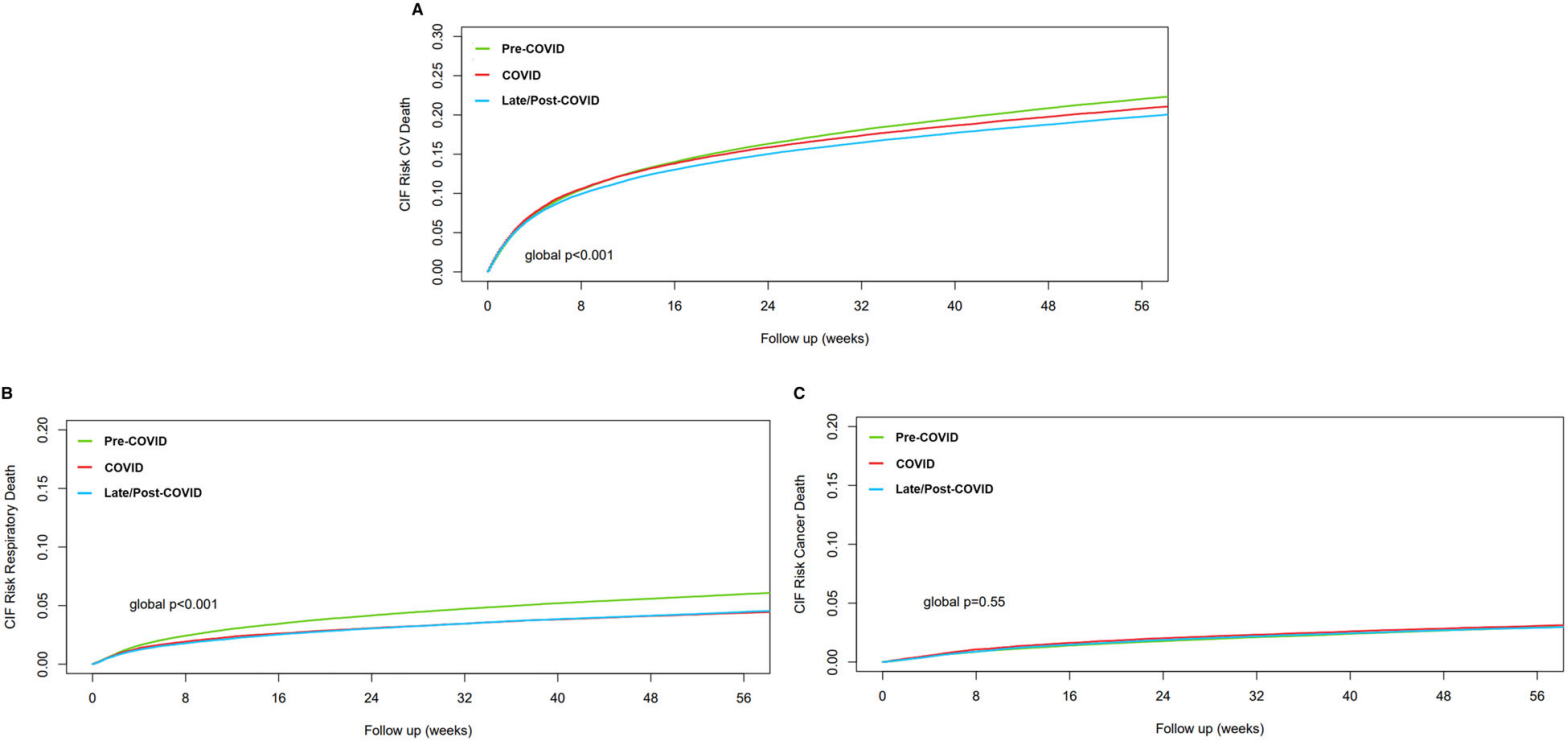
Cumulative incidence function (CIF) for the competing risk of cause‐specific death by admission period in the overall cohort of patients with heart failure. CV, cardiovascular.

Receiving specialist cardiology input during hospitalization was associated with better survival regardless of the period of admission (*p* < 0.001; *Figure* *1A*). For patients with HFrEF receiving specialist cardiology input, longer‐term survival was consistently better compared to those who did not receive specialist cardiology input (*p* < 0.001; *Figure* *1B*). A similar but smaller survival benefit was also seen in those with non‐HFrEF (*p* < 0.001; *Figure* *1C*). Cardiovascular causes accounted for most of the cause‐specific deaths (online supplementary *Figure* *S2*). There was a progressive decrease in cardiovascular (*p* < 0.001; *Figure* *2A*) and respiratory mortality (*p* < 0.001; *Figure* *2B*) over time, while cancer‐related deaths remained stable (*p* = 0.55; *Figure* *2C*).

Compared to pre‐COVID, in‐hospital mortality was similar during COVID (odds ratio [OR] 0.98, 95% CI 0.93–1.02, *p* = 0.20) but lower late/post‐COVID (OR 0.87, 95% CI 0.83–0.91, *p* < 0.001).

### Adjusted analysis

**Figure 3:**
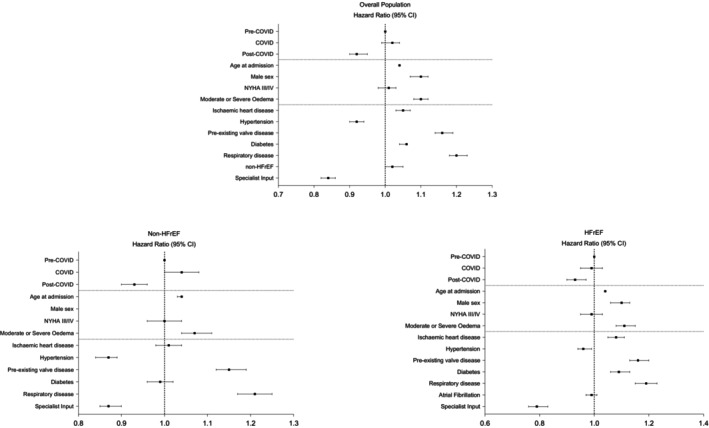
Forest plot based on the results of the multivariable analysis in the overall population (*upper panel*), in non‐heart failure with reduced ejection fraction (HFrEF) (*lower left panel*) and in the HFrEF population (*lower right panel*). CI, confidence interval; NYHA, New York Heart Association.

After adjusting for demographic and clinical characteristics (online supplementary *Figure* *S3*), compared to pre‐COVID, being admitted late/post‐COVID was associated with better long‐term survival (hazard ratio [HR] 0.92, 95% CI 0.90–0.95, *p* < 0.001) driven mainly by a lower all‐cause mortality in the HFrEF cohort (HR 0.93, 95% CI 0.90–0.95, *p* < 0.001) (*Figure* *3*). In contrast, for patients with non‐HFrEF, being admitted during COVID was associated with higher long‐term mortality (HR 1.04, 95% CI 1.00–1.08, *p* < 0.001) while being admitted late/post‐COVID was independently associated with lower mortality compared to pre‐COVID (HR 0.93, 95% CI 0.90–0.97, *p* < 0.001). Receiving specialized cardiology input during hospitalization was associated with lower mortality (HR 0.84, 95% CI 0.82–0.86, *p* < 0.001) both for HFrEF (HR 0.79, 95% CI 0.77–0.82, *p* < 0.001) and for non‐HFrEF (HR 0.87, 95% CI 0.85–0.90, *p* < 0.001).

### Sensitivity analyses

The results of the main analysis are also confirmed in the sensitivity analyses reclassifying the definition of HF (online supplementary *Tables* *S1* and *S2* and *Figures* *S4* and *S5*), excluding patients enrolled in 2022 (online supplementary *Figure* *S6*) and using the imputed dataset (online supplementary *Table* *S3*). Measures of quality of care were maintained in the month‐by‐month analysis within the COVID year (online supplementary *Table* *S4*), and also when the main lockdown measures were analysed (online supplementary *Table* *S5*).

## Discussion

This is the first nationwide analysis to investigate the changes that occurred to the quality of care for patients hospitalized because of HF during the COVID‐19 pandemic. We present data from the largest national registry recording outcomes from a universal healthcare system. We have demonstrated that overall, during the entire COVID‐19 pandemic, metrics outlining the quality of care, such as patients receiving specialist cardiology input, prescription rates for pharmacological therapy and follow‐up arrangements, were maintained in England. Not receiving specialist cardiology care was independently associated with adverse outcomes at each time point.

There were fewer admissions with HF during the pandemic, with a rebound thereafter. Although at a local level we reported that patients admitted during COVID‐19 were generally sicker with a higher in‐hospital mortality compared to previous years,^4^, ^10^, ^12^, ^27^ nationally the baseline characteristics of patients admitted during COVID‐19 appeared similar to those admitted in the two preceding and subsequent years. Two‐thirds of patients were admitted with severe signs and symptoms of HF. Interestingly, the proportion of patients with ischaemic heart disease fell slightly over time, whilst the proportion with hypertension increased. Comorbidity profiles were similar across the three different periods. There are many potential reasons for the discrepancy between local reports and nationwide data including the smaller numbers reported locally, single centres having different pathways of care, as well as the geographical variations in the peaks of the waves of COVID‐19 occurring within a single country.

Although the reconfiguration of healthcare systems may have disrupted the provision of care for patients with cardiovascular disease, our analysis shows that specialist care for HF for those who were admitted to hospital was maintained nationally during the pandemic. Indeed, a progressively greater proportion of patients received specialized cardiology input, regardless of the HF phenotype. Overall, the length of stay was shorter during COVID‐19, with a higher rate of prescribing of pharmacological therapy for HF at discharge than before the pandemic.

The long‐term survival of patients with HF admitted during and in the late phase/after the COVID‐19 pandemic was better compared to the previous period. Although in‐hospital mortality was similar for the three periods, long‐term mortality was significantly better in the late/post COVID period than in previous years. The similar in‐hospital mortality rates may reflect the fact that specialist cardiology care was maintained, where possible, across the country. Indeed, patients receiving specialised cardiology input during hospitalization had more favourable outcomes throughout the pandemic. Conversely, worse outcomes experienced in the early discharge phase during COVID may be explained by changes in community services, difficulties in accessing post‐discharge specialized care and, perhaps, COVID‐related deaths.

Quality of care for several cardiovascular and non‐cardiovascular chronic conditions was described during the pandemic. Cancer diagnosis and treatment were delayed.^28^ Patients with respiratory illness living in lockdown‐impacted regions had difficulties accessing both primary and secondary care.^29^ For patients with cardiovascular conditions, mixed results are reported on the implication of the reconfiguration of care on both in‐hospital procedures and cardiovascular mortality.^30^ However, the improved long‐term mortality rates, especially for patients with HFrEF, may reflect the relative maintenance of specialized cardiology care and subsequent higher rates of prescription of medical therapy at discharge, compared to the past. As previously reported, specialized cardiology care is associated with higher rates of GRMT and lower mortality.^31^ In our analysis, over time, a trend towards a higher rate of prescriptions of GRMT was observed for patients with HFrEF.

Given the time frame of this analysis, it was not possible to investigate the role of important new HF medications, such as sodium–glucose cotransporter 2 inhibitors (SGLT2i). However, it is reasonable to assume that the introduction of this class of drugs might have contributed partially to the observed results (the latest NICOR report from 2022/23 reports the use of SGLT2i in 59% of HFrEF patients)^32^. Furthermore, the vaccination rollout may also have impacted the overall reconfiguration of care and the outcomes of HF patients. The COVID‐19 vaccination rollout, which reduced the incidence and severity of COVID‐19 infections, may have had an impact on our results as it allowed a gradual resumption of more ‘normal’ healthcare pathways. However, further studies are needed to confirm this hypothesis.

This analysis suggests that specialized cardiology care might improve outcomes for patients across the entire LVEF spectrum. Although no specific disease‐modifying treatments for non‐HFrEF were available at the time of the study, more accurate diagnosis, investigation, and treatment of the heterogeneous causes and comorbidities might have improved outcomes. Our finding that better care of both HFrEF and non‐HFrEF patients is associated with better outcomes over time is underscored by the progressive reduction in cardiovascular death during the study. Preservation of specialist care may have led to more initiation and optimization of medical therapy peri‐discharge, in line with recent clinical trials.^33^ These results highlight the important role of specialized care for all patients with HF regardless of the LVEF phenotypes. Even during dramatic times, it is important for healthcare systems to be resilient and maintain specialized care for patients with HF, as this has a substantial prognostic impact.

### Limitations

This study has the limitations of registry‐based research. Patients were not randomized to specialist cardiology care or not. We cannot rule out the possibility of unknown/unmeasured confounders or other biases contributing to the finding that those not receiving specialist care have worse outcomes. The same limitation is pertinent to the prescription of GRMT. We cannot ascertain a causal relationship, but only demonstrate an association with the observed changes and the long‐term outcomes. However, since it is mandatory to report >70% of all discharges coded as HF hospitalizations in England and Wales into the NHFA audit, which represents the largest national HF registry, the risk of selection biases should be small increasing the validity and generalizability of the results. There is also the potential for missing data having an impact on the analysis. To mitigate this, variables with significant missingness (i.e. more than 10% missing) were removed from all multivariable models. Lastly, the specific phenotype and the details of any valve interventions were not known for all patients.

## Conclusions

Despite the significant disruption to clinical care during COVID‐19, the characteristics of patients admitted because of HF during the COVID‐19 year were similar to the preceding and subsequent years. During hospitalization, an increasing proportion of admitted patients received specialist cardiology care. This was associated with higher rates of prescribing for GRMT at discharge, especially for HFrEF. Long‐term outcomes improved over time, driven by a reduction in cardiovascular mortality. Specialized cardiology care was associated with more favourable outcomes across the entire LVEF spectrum.

## Supporting information


**Appendix S1.** Supporting information.
