# Supplementary material for: A nationwide, population‐based study on specialized care for acute heart failure throughout the COVID‐19 pandemic
Source: Eur J Heart Fail. 2024 Jun 4;26(7):1574–84. doi: 10.1002/ejhf.3306 (PMC11613816; doi:10.1002/ejhf.3306)

**Supplementary Materials**

**Supplementary methods**

**ICD codes used for HF diagnosis.**

The National Heart Failure Audit collects data on all patients with an hospital admission with a diagnosis of heart failure in the primary diagnostic position.

Events in the audit are compared with HF episodes codes in the first diagnostic position by Hospital Episode Statistics (HES)

The following ICD-10 codes in the first diagnostic position are used to define the HF hospitalisation to be included in the audit:

I11.0 Hypertensive heart disease with (congestive) heart failure

I25.5 Ischaemic cardiomyopathy

I42.0 Dilated cardiomyopathy

I42.9 Cardiomyopathy, unspecified

I50.0 Congestive heart failure

I50.1 Left ventricular failure

I50.9 Heart failure, unspecified

**Data cleaning process**

To provide a cohort of patients with confirmed diagnosis of HF, we have employed a validation algorithm used by the NICOR. We have excluded patients with unknown or unavailable echocardiograms or with an echocardiogram reported as normal without evidence of atrial fibrillation or atrial flutter.

We have removed patients with the following exclusion criteria:

ICD-10 code for heart failure +

Age less than 18 years

Duplicate Records

Records with a missing or invalid hospital identifier

Date of admission or discharge unknown

Date of discharge preceding the date of admission

Admission and discharge within 24 hrs

**Definition of Heart Failure**

Given the changes in definition of HF across the five years of data collection, we have used a broader definition of HF for the primary analysis (either HFrEF or non-HFrEF).

The main analysis has been conducted using a more stringent definition of HF with reduced ejection Fraction used in version 4 of the NHFA dataset, in which patients in the HFrEF group were classified by LVEF ≤40%. Accordingly, we have classified those with presenting LVEF >40% as having non-HFrEF. Thus, patients with LVEF between 41% and 49% present in the version 5 of NHFA dataset were categorised as non-HFrEF.

However, we have conducted a sensitivity analysis including in the HFrEF cohort patients with systolic dysfunction defined as LVEF ≤40% from Version 4 of the NHFA and LVEF <50% from Version 5 of the NHFA (including, therefore, patients with HF and mildly reduced Ejection Fraction into the HFrEF group).

The echocardiographic definitions of HF included in the National Heart Failure Audit are as follows:

NHFA Version 4:

HFrEF: Presence in the reporting of moderate or severe left ventricular systolic dysfunction (e.g a left ventricular ejection fraction (LVEF) ≤40% conventionally measured or eyeball method of assessment).

Non-HFrEF: Absence of left ventricular systolic dysfunction (i.e. LVEF > 40%) and one (or more) of the following:

- - - Moderate or severe left ventricular hypertrophy reported on an imaging test (e.g LV posterior wall dimension in diastole >1.3cm and/or septal dimension >1.3cm) and/or
    - Moderate or severe stenosis or regurgitation on imaging, or an operative valve replacement/repair and/or
    - Moderate or severe diastolic dysfunction and/or
    - Enlarged left atrium.

NHFA Version 5:

HFrEF: Presence in the reporting of:

- - - Moderate or severe left ventricular systolic dysfunction (e.g. a left ventricular ejection fraction (LVEF) ≤40% or eyeball method of assessment).
    - Mild systolic dysfunction (e.g. a left ventricular ejection fraction(LVEF 41% - 49% or eyeball method of assessment).

Non-HFrEF: Absence of left ventricular systolic dysfunction (i.e. LVEF ≥50%) and one (or more) of the following:

- - - Moderate or severe left ventricular hypertrophy reported on an imaging test (e.g. LV posterior wall dimension in diastole >1.3cm and/or septal dimension >1.3cm) and/or
    - Moderate or severe stenosis or regurgitation on imaging, or an operative valve replacement/repair and/or
    - Moderate or severe diastolic dysfunction and/or
    - Enlarged left atrium and/or
    - Cor Pulmonale/Right Heart failure due to lung disease.

Supplementary Table 1. Characteristics of the cohort with HFrEF with LVEF ≤50%.

|  |  | Pre-COVID | | COVID | | Late/Post-COVID | |
| --- | --- | --- | --- | --- | --- | --- | --- |
| n of patients | | 57,280 | | 23,285 | | 39,910 | |
|  | Male sex | 36,700 | 64% | 14,910 | 64% | 25,770 | 65% |
|  | Age at admission | 79 | 70;86 | 78 | 68;86 | 78 | 67;86 |
| ethnicity | White | 25,650 | 86% | 10,330 | 89% | 17,920 | 90% |
|  | Black | 840 | 3% | 325 | 3% | 660 | 3% |
|  | Asian | 1,840 | 6% | 560 | 5% | 120 | 1% |
|  | Others | 1,430 | 5% | 440 | 4% | 1,265 | 6% |
|  | NYHA III/IV | 42,455 | 77% | 17,075 | 77% | 29,075 | 76% |
|  | Moderate or Severe Oedema | 26,420 | 49% | 10,440 | 48% | 17,300 | 46% |
|  | IHD | 24,250 | 44% | 8,845 | 40% | 14,130 | 37% |
|  | Pre-existing valve disease | 15,080 | 27% | 5,690 | 25% | 9,810 | 24% |
|  | Hypertension | 29,220 | 52% | 12,160 | 53% | 21,220 | 54% |
|  | Diabetes | 18,940 | 34% | 7,350 | 32% | 12,630 | 32% |
|  | Respiratory disease | 13,130 | 24% | 5,250 | 23% | 8,650 | 22% |
| ECG | Sinus | 24,490 | 46% | 9,900 | 46% | 18,280 | 49% |
|  | Atrial Fibrillation | 22,240 | 42% | 9,180 | 42% | 15,140 | 41% |
|  | Other | 6,640 | 12% | 2,570 | 12% | 3,750 | 10% |
|  | Heart Rate | 86 | 72;104 | 89 | 74;107 | 88 | 74;106 |
|  | Creatinine at admission | 107 | 83;146 | 105 | 82;141 | 103 | 81;139 |
|  | eGFR (CKD-EPI) | 51 | 34;70 | 53 | 36;72 | 55 | 37;75 |
|  | Systolic BP | 128 | 112;147 | 130 | 113;149 | 130 | 114;149 |
|  | Potassium | 4.2 | 3.9;4.6 | 4.2 | 3.8;4.6 | 4.2 | 3.9;4.6 |
| Discharge Medications | ACEi/ARBs/ARNI | 30,350 | 70% | 12,920 | 72% | 23,650 | 73% |
|  | Beta-Blocker | 36,980 | 84% | 15,520 | 85% | 27,875 | 86% |
|  | Diuretics | 39,920 | 90% | 16,000 | 89% | 27,700 | 87% |
|  | MRAs | 19,685 | 51% | 8,470 | 54% | 16,775 | 57% |
|  | Nurse Follow-up | 33,130 | 73% | 12,975 | 69% | 14,355 | 43% |
|  | Cardiology Follow-up | 25,775 | 58% | 9,910 | 54% | 13,240 | 40% |
|  | Specialist input | 49,740 | 86% | 20,560 | 88% | 36,120 | 91% |
|  | Follow-up (weeks) | 122 | 23;196 | 110 | 21;131 | 44 | 20;71 |
|  | Length of stay (days) | 8 | 4;15 | 7 | 4;13 | 8 | 4;14 |

Supplementary Table 2. Characteristics of the cohort with non-HFrEF and LVEF >50%

| \|  \|  \| Pre-COVID \| \| COVID \| \| Late/Post-COVID \| \| \| --- \| --- \| --- \| --- \| --- \| --- \| --- \| --- \| \| n of patients \|  \| 57,280 \| \| 23,280 \| \| 39,910 \| \| \|  \| Male sex \| 21,000 \| 44% \| 9,410 \| 45% \| 18,185 \| 47% \| \|  \| Age at admission \| 86 \| 76;89% \| 83 \| 76;86% \| 83 \| 76;89% \| \| Ethnicity \| White \| 19,825 \| 86% \| 9,600 \| 89% \| 17,960 \| 87% \| \| Black \| 630 \| 3% \| 255 \| 2% \| 510 \| 2% \| \| Asian \| 1,435 \| 6% \| 540 \| 5% \| 1,195 \| 6% \| \| Others \| 1,120 \| 5% \| 350 \| 3% \| 950 \| 5% \| \|  \| NYHA III/IV \| 35,325 \| 77% \| 15,405 \| 77% \| 28,480 \| 76% \| \|  \| Moderate or Severe Oedema \| 26,000 \| 49% \| 11,370 \| 48% \| 21,355 \| 46% \| \|  \| IHD \| 15,190 \| 33% \| 6,390 \| 32% \| 11,100 \| 30% \| \|  \| Pre-existing valve disease \| 15,185 \| 33% \| 6,520 \| 33% \| 11,325 \| 30% \| \|  \| Hypertension \| 28,490 \| 61% \| 12,890 \| 63% \| 24,430 \| 64% \| \|  \| Diabetes \| 15,600 \| 34% \| 6,730 \| 33% \| 12,775 \| 33% \| \|  \| Respiratory disease \| 14,220 \| 31% \| 5,295 \| 26% \| 10,045 \| 26% \| \|  \| Heart Rate \| 81 \| 69;97 \| 82 \| 70;98 \| 82 \| 69;96 \| \|  \| Creatinine at admission \| 104 \| 80;141 \| 103 \| 79;141 \| 102 \| 79;139 \| \|  \| eGFR (CKD-EPI) \| 49 \| 34;67 \| 50 \| 33;67 \| 50 \| 34;68 \| \|  \| Systolic BP \| 135 \| 118;154 \| 137 \| 119;156 \| 136 \| 119;156 \| \|  \| Potassium \| 4.2 \| 3.8;4.6 \| 4.1 \| 3.8;4.5 \| 4.1 \| 3.8;4.5 \| \| ECG \| Sinus \| 28,130 \| 61% \| 5,985 \| 30% \| 12,050 \| 32% \| \| Atrial Fibrillation \| 3,255 \| 57% \| 12,520 \| 63% \| 2,850 \| 61% \| \| Other \| 6,640 \| 12% \| 1,440 \| 7% \| 2,290 \| 6% \| \|  \| Predominant Valve disease \| 13,080 \| 28% \| 6,865 \| 33% \| 11,265 \| 29% \| \| Discharge Medications \| ACEi/ARBs/ARNI \| 15,440 \| 53% \| 6,770 \| 53% \| 13,840 \| 53% \| \| Beta-Blocker \| 23,580 \| 73% \| 10,560 \| 74% \| 21,160 \| 75% \| \| Diuretics \| 34,370 \| 93% \| 15,160 \| 93% \| 29,395 \| 93% \| \| MRAs \| 8,060 \| 32% \| 3,550 \| 33% \| 7,390 \| 34% \| \|  \| Nurse Follow-up \| 13,300 \| 36% \| 5,340 \| 33% \| 6,840 \| 21% \| \|  \| Cardiology Follow-up \| 13,890 \| 37% \| 5,640 \| 35% \| 8,750 \| 28% \| \|  \| Specialist input \| 33,070 \| 70% \| 14,540 \| 70% \| 28,750 \| 74% \| \|  \| Follow-up (weeks) \| 90 \| 20;181% \| 85 \| 15;125% \| 42 \| 18;71% \| \|  \| Length of stay (days) \| 7 \| 3;14% \| 7 \| 3;13 \| 8 \| 3;13 \| |  |  |  |  |  |
| --- | --- | --- | --- | --- | --- | --- | --- | --- | --- | --- | --- | --- | --- | --- | --- | --- | --- | --- | --- | --- | --- | --- | --- | --- | --- | --- | --- | --- | --- | --- | --- | --- | --- | --- | --- | --- | --- | --- | --- | --- | --- | --- | --- | --- | --- | --- | --- | --- | --- | --- | --- | --- | --- | --- | --- | --- | --- | --- | --- | --- | --- | --- | --- | --- | --- | --- | --- | --- | --- | --- | --- | --- | --- | --- | --- | --- | --- | --- | --- | --- | --- | --- | --- | --- | --- | --- | --- | --- | --- | --- | --- | --- | --- | --- | --- | --- | --- | --- | --- | --- | --- | --- | --- | --- | --- | --- | --- | --- | --- | --- | --- | --- | --- | --- | --- | --- | --- | --- | --- | --- | --- | --- | --- | --- | --- | --- | --- | --- | --- | --- | --- | --- | --- | --- | --- | --- | --- | --- | --- | --- | --- | --- | --- | --- | --- | --- | --- | --- | --- | --- | --- | --- | --- | --- | --- | --- | --- | --- | --- | --- | --- | --- | --- | --- | --- | --- | --- | --- | --- | --- | --- | --- | --- | --- | --- | --- | --- | --- | --- | --- | --- | --- | --- | --- | --- | --- | --- | --- | --- | --- | --- | --- | --- | --- | --- | --- | --- | --- | --- | --- | --- | --- | --- | --- | --- | --- | --- | --- | --- | --- | --- | --- | --- | --- | --- | --- | --- | --- | --- | --- | --- | --- | --- | --- | --- | --- | --- | --- | --- | --- | --- | --- | --- | --- | --- | --- | --- | --- | --- | --- | --- | --- | --- | --- | --- | --- | --- | --- | --- | --- | --- | --- | --- | --- | --- | --- | --- | --- | --- | --- | --- |

Supplementary Table 3. Comparison between complete case multivariable model and using multiple imputation to handle missing data.

| Variable | | Complete case analysis n=163,650 | | | |  | Imputed dataset n=227,250 | | | |
| --- | --- | --- | --- | --- | --- | --- | --- | --- | --- | --- |
|  |  | HR | 95% CI | | p-value |  | HR | 95% CI | | p-value |
| COVID periods | Pre-COVID | ref |  |  | <0.001 |  | ref |  |  | <0.001 |
|  | COVID | 1.02 | 0.99 | 1.04 |  |  | 1.02 | 1.01 | 1.04 |  |
|  | Post-COVID | 0.92 | 0.90 | 0.95 |  |  | 0.94 | 0.93 | 0.96 |  |
|  | Male sex | 1.10 | 1.07 | 1.12 | <0.001 |  | 1.10 | 1.08 | 1.11 | <0.001 |
|  | Age at admission | 1.04 | 1.04 | 1.04 | <0.001 |  | 1.04 | 1.04 | 1.04 | <0.001 |
| ethnicity | Ethnic minorities | 0.82 | 0.79 | 0.85 | <0.001 |  | 0.86 | 0.83 | 0.89 | <0.001 |
|  | NYHA III/IV | 1.01 | 0.98 | 1.03 | 0.9 |  | 1.04 | 1.02 | 1.05 | <0.001 |
|  | Moderate or Severe Oedema | 1.10 | 1.08 | 1.12 | <0.001 |  | 1.12 | 1.11 | 1.14 | <0.001 |
|  | IHD | 1.05 | 1.03 | 1.07 | <0.001 |  | 1.07 | 1.06 | 1.08 | <0.001 |
|  | Pre-existing valve disease | 1.16 | 1.14 | 1.19 | <0.001 |  | 1.14 | 1.13 | 1.16 | <0.001 |
|  | HTN | 0.92 | 0.90 | 0.94 | <0.001 |  | 0.90 | 0.89 | 0.91 | <0.001 |
|  | Diabetes | 1.04 | 1.06 | 1.06 | <0.001 |  | 1.06 | 1.05 | 1.07 | <0.001 |
|  | Respiratory diseasae | 1.20 | 1.18 | 1.23 | <0.001 |  | 1.20 | 1.18 | 1.21 | <0.001 |
|  | Atrial Fibrillation | 0.97 | 0.95 | 1.00 | 0.03 |  | 0.99 | 0.98 | 1.00 | 0.2 |
|  | HFnEF vs HFrEF | 1.02 | 1.00 | 1.05 | 0.048 |  | 1.02 | 1.00 | 1.03 | 0.01 |
|  | Heart Rate | 1.00 | 1.00 | 1.00 | 0.2 |  | 1.00 | 1.00 | 1.00 | 0.03 |
| Systolic Blood Pressure at admission | <105 | 1.43 | 1.38 | 1.49 | <0.001 |  | 1.15 | 1.13 | 1.17 | <0.001 |
|  | 105-115 | 1.17 | 1.13 | 1.22 |  |  | 1.16 | 1.13 | 1.18 |  |
|  | 115-123 | ref |  |  |  |  | ref |  |  |  |
|  | 123-132 | 0.93 | 0.90 | 0.97 |  |  | 0.93 | 0.91 | 0.95 |  |
|  | 132-140 | 0.85 | 0.81 | 0.89 |  |  | 0.87 | 0.85 | 0.89 |  |
|  | 140-151 | 0.81 | 0.78 | 0.84 |  |  | 0.82 | 0.8 | 0.84 |  |
|  | 151-166 | 0.74 | 0.71 | 0.77 |  |  | 0.76 | 0.74 | 0.78 |  |
|  | >166 | 0.67 | 0.64 | 0.70 |  |  | 0.70 | 0.69 | 0.72 |  |
| Potassium at discharge | <3.5 | 1.35 | 1.30 | 1.41 | <0.001 |  | 1.30 | 1.27 | 1.33 | <0.001 |
|  | 3.5-4 | 1.1 | 1.07 | 1.13 |  |  | 1.09 | 1.07 | 1.11 |  |
|  | 4-4.5 | ref |  |  |  |  | ref |  |  |  |
|  | 4.5-5 | 1.04 | 1.01 | 1.07 |  |  | 1.03 | 1.01 | 1.05 |  |
|  | 5-5.5 | 1.32 | 1.26 | 1.37 |  |  | 1.25 | 1.22 | 1.28 |  |
|  | 5.5-6 | 1.95 | 1.83 | 2.09 |  |  | 1.77 | 1.72 | 1.83 |  |
|  | >6 | 3.83 | 3.49 | 4.21 |  |  | 2.74 | 2.63 | 2.84 |  |
| Sodium at discharge | <125 | 2.07 | 1.92 | 2.23 | <0.001 |  | 0.88 | 0.85 | 0.90 | <0.001 |
|  | 125-135 | 1.33 | 1.30 | 1.37 |  |  | 1.30 | 1.28 | 1.32 |  |
|  | 135-145 | ref |  |  |  |  | ref |  |  |  |
|  | >145 | 2.5 | 2.38 | 2.62 |  |  | 2.48 | 2.41 | 2.55 |  |
| Urea at Discharge | <7.8 | ref |  |  | <0.001 |  | ref |  |  | <0.001 |
|  | 7.8-12 | 1.21 | 1.18 | 1.25 |  |  | 1.21 | 1.19 | 1.23 |  |
|  | 12-20 | 1.64 | 1.59 | 1.7 |  |  | 1.64 | 1.6 | 1.67 |  |
|  | 20-33 | 2.36 | 2.27 | 2.46 |  |  | 2.29 | 2.23 | 2.35 |  |
|  | >33 | 2.35 | 2.24 | 2.47 |  |  | 2.16 | 2.1 | 2.22 |  |
|  | eGFR | 1.00 | 1.00 | 1.00 | <0.001 |  | 1.00 | 1.00 | 1.00 | <0.001 |
|  | Specialist input | 0.84 | 0.82 | 0.86 | <0.001 |  | 0.85 | 0.84 | 0.86 | <0.001 |

Supplementary table 4. Characteristics of patients enrolled during COVID in the month-by-month analysis.

| **n of patients** |  | **January, N = 4,560** | **February, N = 4,270** | **March, N = 2,890** | **April, N = 2,480** | **May, N = 3,820** | **June, N = 4,200** | **July, N = 4,080** | **August, N = 3,540** | **September, N = 3,730** | **October, N = 3,750** | **November, N = 3,380** | **December, N = 3,320** |
| --- | --- | --- | --- | --- | --- | --- | --- | --- | --- | --- | --- | --- | --- |
|  | Male sex | 2,580 (57%) | 2,310 (54%) | 1,620 (56%) | 1,400 (56%) | 2,120 (56%) | 2,260 (54%) | 2,230 (55%) | 1,940 (55%) | 2,030 (54%) | 2,110 (56%) | 1,900 (56%) | 1,830 (55%) |
|  | Age at admission | 81 [72, 87] | 82 [73, 88] | 82 [73, 88] | 80 [72, 87] | 81 [72, 87] | 81 [72, 87] | 81 [72, 87] | 81 [72, 87] | 81 [72, 87] | 81 [72, 88] | 81 [72, 88] | 82 [73, 88] |
| **Ethnicity** | White | 2,050 (89%) | 1,920 (89%) | 1,330 (89%) | 1,150 (90%) | 1,670 (89%) | 1,840 (89%) | 1,810 (89%) | 1,540 (87%) | 1,710 (90%) | 1,750 (90%) | 1,630 (89%) | 1,510 (89%) |
|  | Black | 60 (3%) | 60 (3%) | 30 (2%) | 30 (2%) | 50 (3%) | 60 (3%) | 50 (3%) | 50 (3%) | 40 (2%) | 50 (3%) | 50 (3%) | 50 (3%) |
|  | Asian | 110 (5%) | 100 (5%) | 80 (5%) | 60 (3%) | 90 (5%) | 90 (4%) | 110 (4%) | 110 (6%) | 90 (5%) | 90 (5%) | 90 (5%) | 70 (4%) |
|  | Others | 80 (3%) | 70 (3%) | 60 (4%) | 40 (5%) | 70 (4%) | 70(3%) | 70 (3%) | 70 (4%) | 70 (4%) | 50 (3%) | 70 (4%) | 60 (4%) |
|  | NYHA III/IV | 3,400 (78%) | 3,170 (78%) | 2,140 (78%) | 1,840 (79%) | 2,820 (78%) | 3,060 (77%) | 2,990 (77%) | 2,540 (75%) | 2,750 (78%) | 2,760 (77%) | 2,520 (78%) | 2,430 (77%) |
|  | Moderate or Severe Oedema | 2,250 (53%) | 2,130 (54%) | 1,440 (54%) | 1,270 (56%) | 1,980 (56%) | 2,160 (55%) | 2,050 (54%) | 1,680 (52%) | 1,780 (51%) | 1,800 (51%) | 1,640 (52%) | 1,610 (53%) |
|  | IHD | 1,610 (37%) | 1,480 (36%) | 970 (35%) | 830 (34%) | 1,300 (35%) | 1,440 (36%) | 1,390 (35%) | 1,290 (38%) | 1,280 (35%) | 1,350 (37%) | 1,180 (36%) | 1,130 (35%) |
|  | Pre-existing valve disease | 1,270 (29%) | 1,230 (30%) | 800 (29%) | 730 (30%) | 1,070 (29%) | 1,200 (29%) | 1,150 (29%) | 970 (28%) | 1,010 (28%) | 990 (27%) | 870 (27%) | 920 (29%) |
|  | Hypertension | 2,600 (58%) | 2,350 (56%) | 1,630 (57%) | 1,370 (56%) | 2,140 (57%) | 2,400 (58%) | 2,310 (57%) | 2,030 (58%) | 2,170 (59%) | 2,150 (58%) | 1,960 (59%) | 1,940 (59%) |
|  | Diabetes | 1,460 (33%) | 1,400 (33%) | 890 (31%) | 770 (31%) | 1,210 (32%) | 1,330 (32%) | 1,320 (33%) | 1,160 (34%) | 1,210 (33%) | 1,190 (32%) | 1,080 (33%) | 1,070 (33%) |
|  | Respiratory disease | 1,090 (25%) | 1,010 (24%) | 630 (22%) | 580 (24%) | 930 (25%) | 1,030 (25%) | 1,050 (26%) | 850 (25%) | 920 (25%) | 870 (24%) | 830 (25%) | 760 (23%) |
|  | Heart Rate | 85 [71, 101] | 85 [71, 101] | 85 [70, 102] | 88 [73, 106] | 86 [72, 103] | 86 [71, 103] | 86 [72, 103] | 84 [70, 101] | 86 [71, 102] | 86 [71, 103] | 85 [72, 104] | 85 [71, 101] |
|  | Creatinine at admission | 104 [81, 143] | 104 [80, 142] | 104 [82, 140] | 104 [80, 145] | 104 [80, 142] | 103 [80, 139] | 102 [80, 138] | 103 [80, 139] | 105 [80, 142] | 104 [81, 140] | 105 [82, 142] | 105 [81, 143] |
|  | eGFR (CKD-EPI) | 51 [33, 70] | 50 [33, 69] | 51 [35, 70] | 51 [33, 72] | 51 [35, 70] | 52 [36, 70] | 52 [33, 70] | 52 [36, 71] | 51 [34, 69] | 51 [36, 70] | 52 [35, 69] | 51 [34, 68] |
|  | Systolic BP | 131 [115, 150] | 132 [115, 151] | 132 [116, 152] | 133 [115, 153] | 133 [116, 153] | 133 [117, 154] | 133 [116, 152] | 132 [114, 152] | 134 [116, 154] | 134 [117, 154] | 135 [118, 154] | 135 [118, 156] |
|  | Potassium | 4.20 [3.80, 4.60] | 4.20 [3.80, 4.60] | 4.20 [3.80, 4.60] | 4.10 [3.80, 4.50] | 4.10 [3.80, 4.50] | 4.10 [3.80, 4.50] | 4.20 [3.80, 4.50] | 4.10 [3.80, 4.50] | 4.20 [3.80, 4.50] | 4.20 [3.80, 4.60] | 4.20 [3.80, 4.50] | 4.10 [3.80, 4.50] |
|  | Atrial Fibrillation | 2,250 (52%) | 2,180 (54%) | 1,460 (53%) | 1,270 (55%) | 1,860 (52%) | 2,060 (52%) | 1,970 (51%) | 1,640 (49%) | 1,800 (51%) | 1,920 (54%) | 1,660 (51%) | 1,640 (52%) |
|  | HFrEF | 2,490 (55%) | 2,210 (52%) | 1,510 (52%) | 1,340 (54%) | 2,020 (53%) | 2,340 (56%) | 2,170 (53%) | 1,940 (55%) | 2,030 (54%) | 1,950 (52%) | 1,690 (50%) | 1,590 (48%) |
|  | non-HFrEF | 2,070 (45%) | 2,060 (48%) | 1,390 (48%) | 1,140 (46%) | 1,800 (47%) | 1,860 (44%) | 1,910 (47%) | 1,590 (45%) | 1,710 (46%) | 1,800 (48%) | 1,690 (50%) | 1,730 (52%) |
| **Discharge Medications** | RASi/ARNI | 1,970 (63%) | 1,790 (61 | 1,240 (63%) | 1,050 (63%) | 1,750 (65%) | 1,950 (66%) | 1,810 (63%) | 1,670 (65%) | 1,720 (65%) | 1750 (66%) | 1,550 (65%) | 1,420 (64%) |
|  | Beta-Blocker | 2,610 (79%) | 2,490 (79%) | 1,650 (79%) | 1,390 (80%) | 2,290 (80%) | 2,560 (81%) | 2,490 (81%) | 2,140 (79%) | 2,300 (82%) | 2,260 (81%) | 2,030 (81%) | 1,855 (80%) |
|  | Diuretics | 3,130 (90%) | 3,030 (91%) | 2,050 (92%) | 1,690 (91%) | 2,770 (92%) | 3,040 (91%) | 2,940 (91%) | 2,530 (90%) | 2,681 (91%) | 2,700 (92%) | 2,360 (90%) | 2,230 (90%) |
|  | MRAs | 1,210 (45%) | 1,100 (43%) | 750 (44%) | 650 (46%) | 1,080 (47%) | 1,180 (47%) | 1,130 (46%) | 1,040 (46%) | 1,050 (46%) | 1,070 (47%) | 900 (44%) | 860 (46%) |
|  | Nurse Follow-up | 2,070 (58%) | 1,860 (55%) | 1,180 (53%) | 1,040 (55%) | 1,740 (56%) | 1,960 (57%) | 1,780 (54%) | 1,550 (54%) | 1,570 (52%) | 1,390 (46%) | 1,180 (44%) | 980 (39%) |
|  | Cardiology Follow-up | 1,770 (50%) | 1,611 (48%) | 1,000 (45%) | 850 (46%) | 1,380 (45%) | 1,520 (45%) | 1,510 (46%) | 1,280 (45%) | 1,390 (47%) | 1,250 (42%) | 1,060 (40%) | 920 (37%) |
|  | Specialist input | 3,690 (81%) | 3,430 (80%) | 2,210 (77%) | 1,850 (75%) | 2,950 (77%) | 3,340 (80%) | 3,220 (79%) | 2,830 (80%) | 3,050 (82%) | 3,030 (81%) | 2,790 (83%) | 2,690 (81%) |
|  | Follow-up (weeks) | 108 [21, 154] | 100 [19, 150] | 96 [11, 146] | 94 [10, 141] | 109 [22, 137] | 117 [23, 133] | 113 [22, 128] | 122 [22, 124] | 117 [18, 120] | 113 [17, 115] | 109 [15, 111] | 88 [9, 107] |
|  | Length of stay (days) | 7 [3, 14] | 8 [4, 15] | 7 [3, 12] | 6 [3, 12] | 7 [3, 12] | 7 [4, 12] | 7 [4, 13] | 7 [4, 14] | 7 [4, 14] | 8 [4, 14] | 7 [4, 13] | 7 [3, 13] |

Supplementary table 5. Characteristics of patients enrolled during COVID lockdowns.

| **n of patients** |  | **First Lockdown, N=13,550** | **Second lockdown, N=22,020** |
| --- | --- | --- | --- |
|  | Male sex | 7,470 (55%) | 12,320 (56%) |
|  | Age at admission | 80 [72, 88] | 81 [72, 88] |
| **Ethnicity** | White | 6,080 (89%) | 10,080 (89%) |
|  | Black | 170 (2%) | 330 (3%) |
|  | Asian | 330 (5%) | 570 (5%) |
|  | Others | 250 (4%) | 400 (4%) |
|  | NYHA III/IV | 9,990 (78%) | 16,200 (77%) |
|  | Moderate or Severe Oedema | 6,940 (55%) | 10,830 (53%) |
|  | IHD | 4,580 (35%) | 7,430 (35%) |
|  | Pre-existing valve disease | 3,840 (29%) | 5,830 (27%) |
|  | Hypertension | 7,610 (57%) | 12,850 (59%) |
|  | Diabetes | 4,240 (32%) | 6,990 (32%) |
|  | Respiratory disease | 3,210 (24%) | 5,140 (24%) |
|  | Heart Rate | 86 [72, 103] | 86 [71, 103] |
|  | Creatinine at admission | 103 [80, 141] | 104 [81, 141] |
|  | eGFR (CKD-EPI) | 51 [35, 70] | 51 [35, 69] |
|  | Systolic BP | 133 [116, 153] | 135 [117, 154] |
|  | Potassium | 4.10 [3.80, 4.50] | 4.20 [3.80, 4.50] |
|  | Atrial Fibrillation | 6,730 (53%) | 10,952 (52%) |
|  | HFrEF | 7,300 (54%) | 10,760 (49%) |
|  | non-HFrEF | 6,250 (46%) | 11,264 (51%) |
| **Discharge Medications** | RASi/ARNI | 6,060 (64%) | 10,050 (65%) |
|  | Beta-Blocker | 7,990 (80%) | 13,147 (81%) |
|  | Diuretics | 9,670 (92%) | 15,560 (90%) |
|  | MRAs | 3,700 (46%) | 6,150 (46%) |
|  | Nurse Follow-up | 5,990 (55%) | 6,790 (38%) |
|  | Cardiology Follow-up | 4,810 (45%) | 6,610 (38%) |
|  | Specialist input | 10,480 (77%) | 18,050 (82%) |
|  | Follow-up (weeks) | 107 [18, 137] | 93 [17, 103] |
|  | Length of stay (days) | 7 [3, 12] | 7 [4, 13] |

Supplementary Figure 1.

Panel A: Weekly Admissions because of Acute Heart Failure across the COVID Pandemic

Panel B: Monthly admissions because of Acute Heart Failure From 2018 to 2022


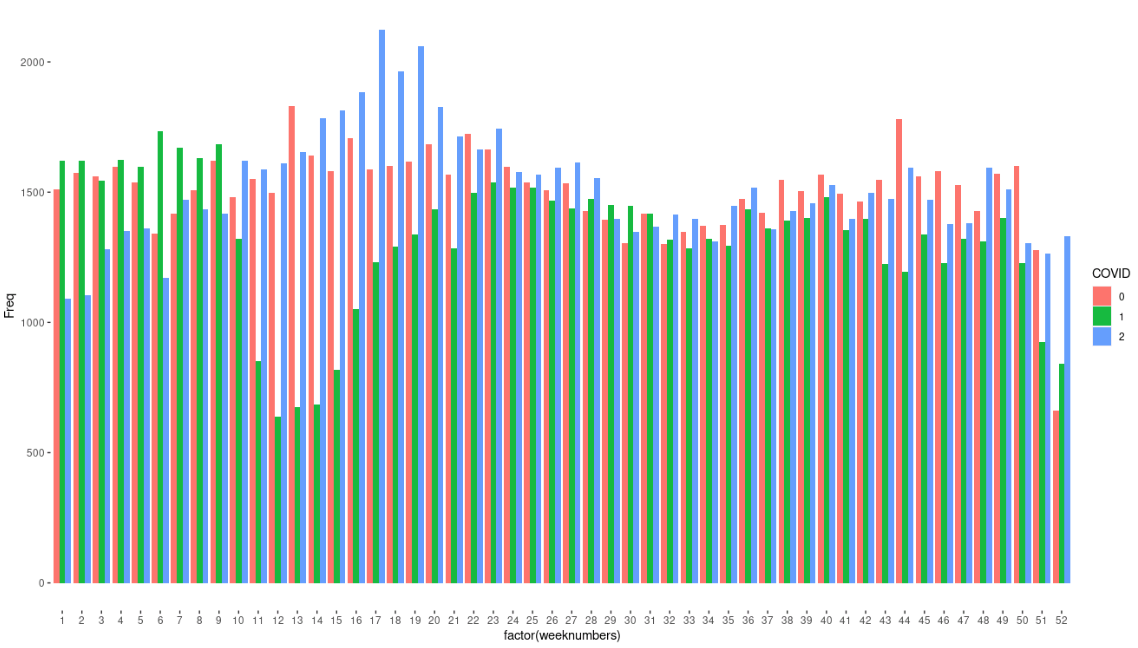


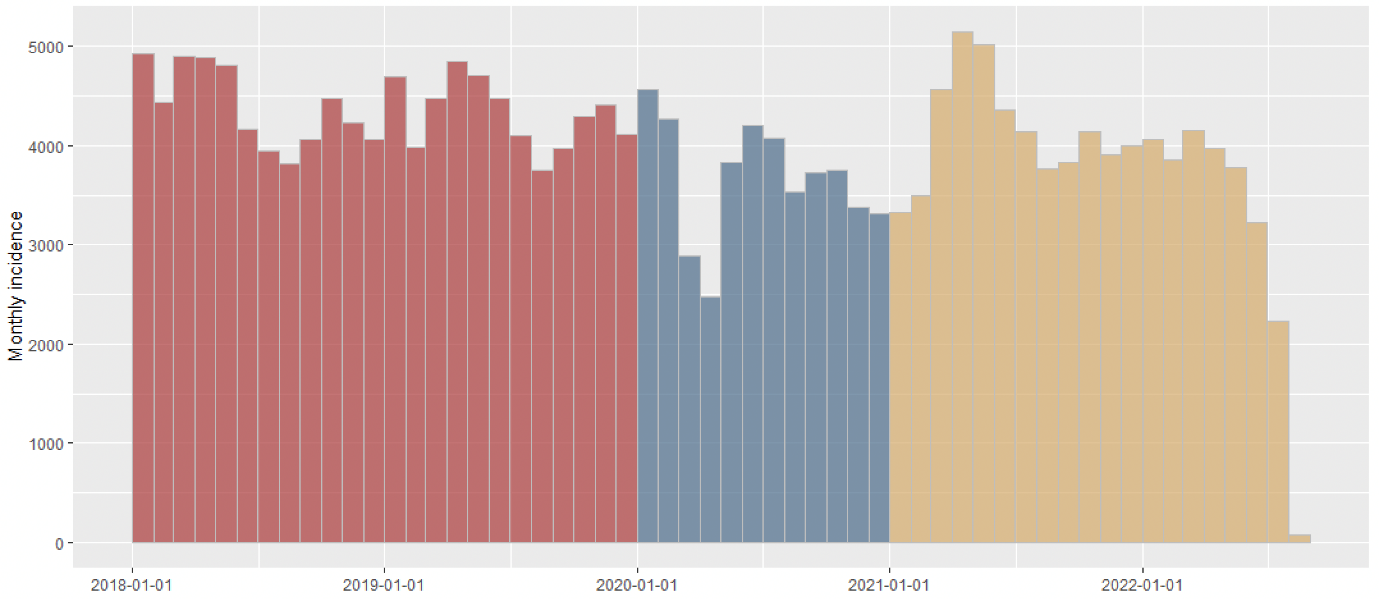


Legend: 0= Pre-COVID; 1= COVID; 2= Late/Post-COVID.

Supplementary Figure 2. Cause specific deaths for patients with HF.

Supplementary Figure 3. Forest plot based on the results of the Cox-Proportional multivariable analysis of the factors associated with overall survival in patients with Heart Failure (Left Panel), in patients with HFrEF (Centre Panel) and in patients with non-HFrEF (Right Panel).

Supplementary Figure 4. Sensitivity analysis on patients with LVEF ≤ or > 50%


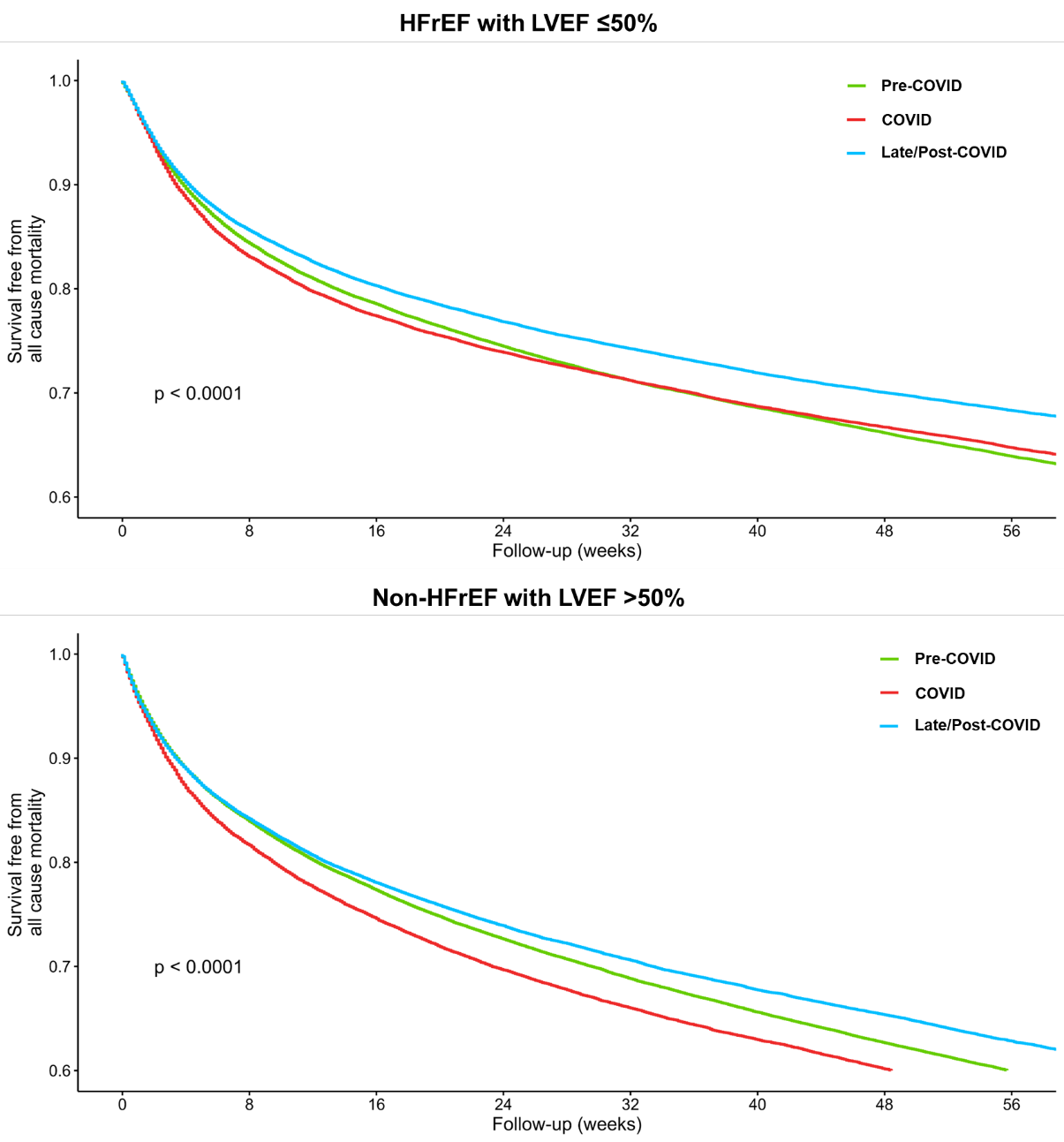


Supplementary Figure 5. Sensitivity analysis on patients with LVEF ≤ or > 50% and according to specialist input.


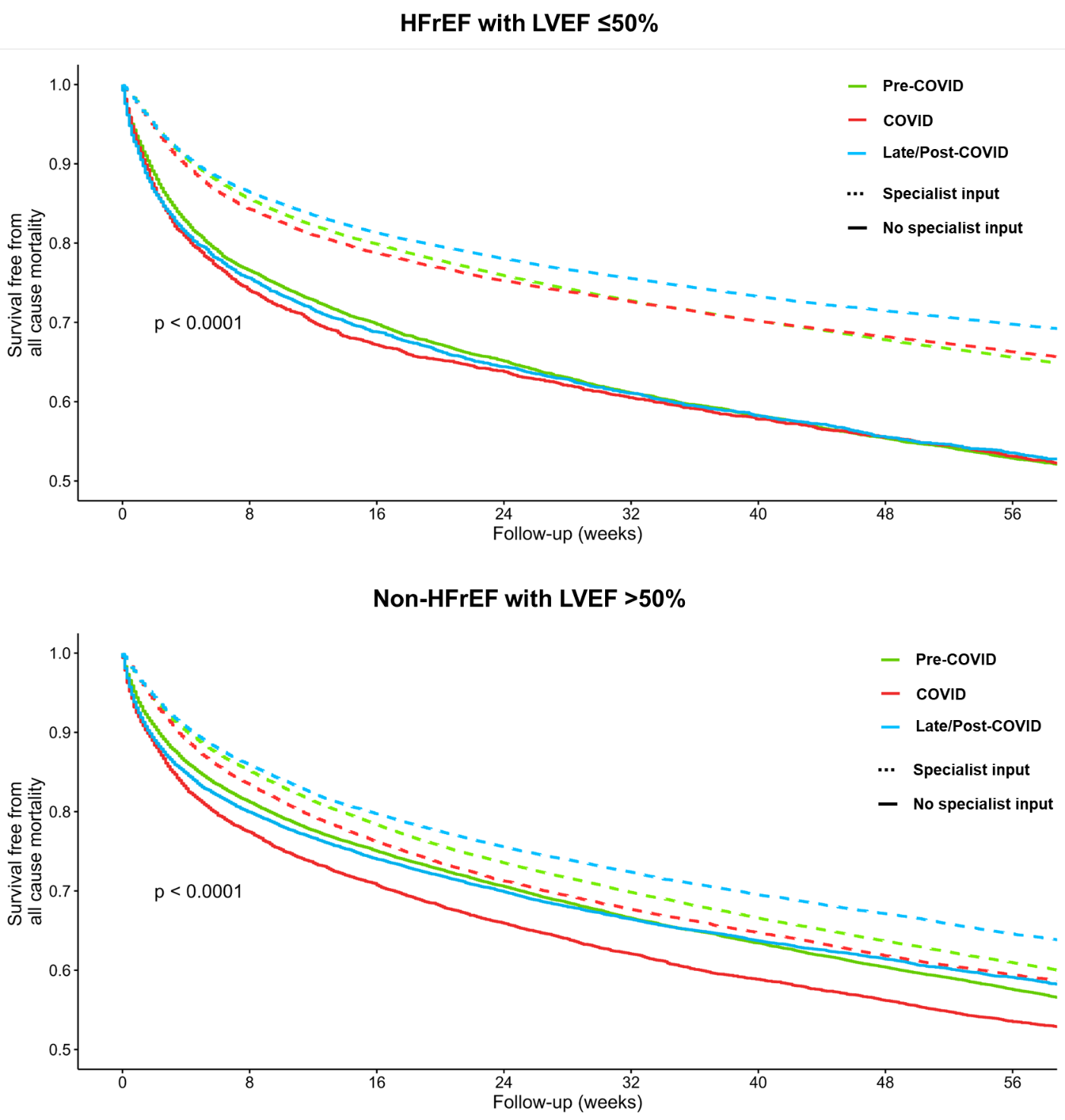


Supplementary Figure 6. Sensitivity analysis excluding patients enrolled in 2022


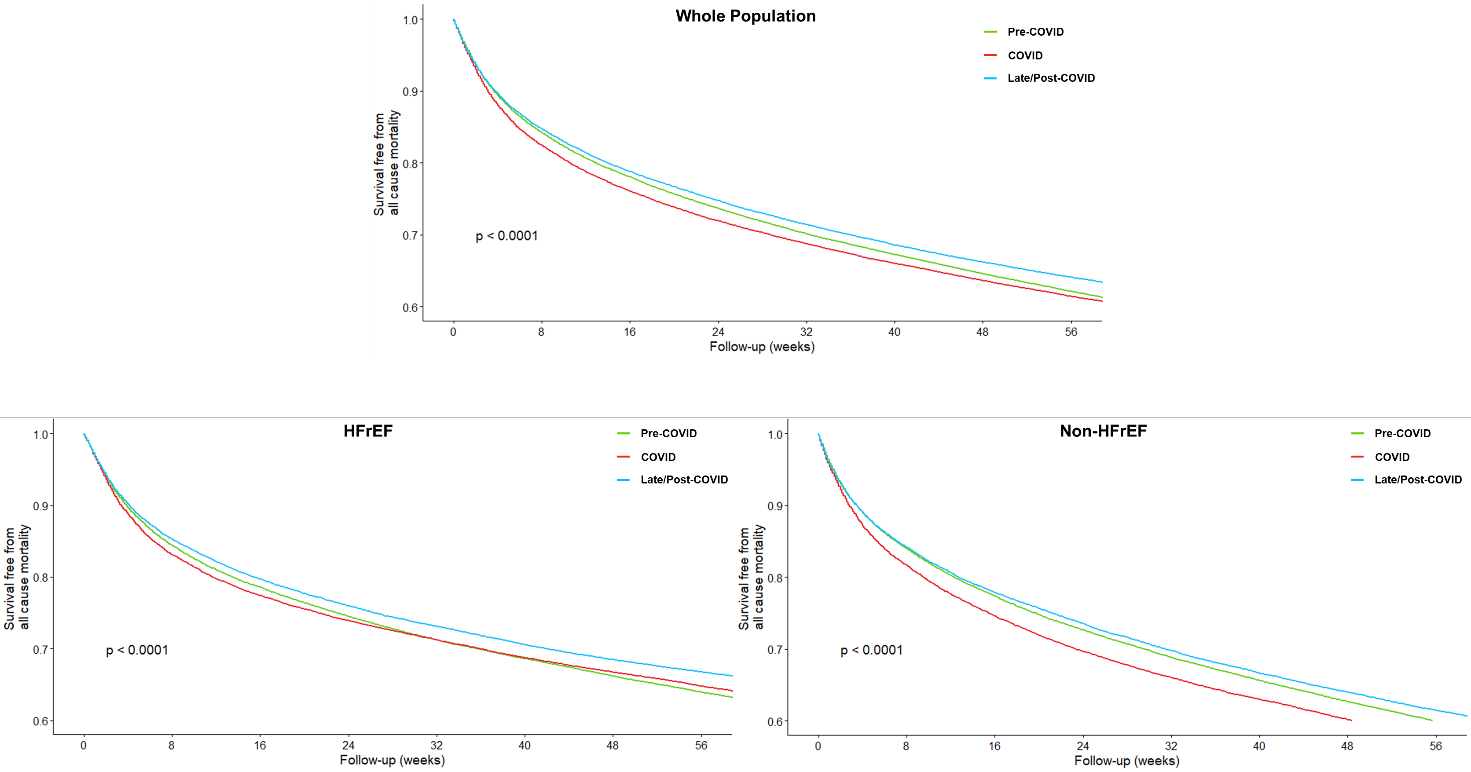

Supplement: Supplementary file 1 — Appendix S1. Supporting information. [file EJHF-26-1574-s001.docx]
